# Seasonal Pattern in the Diagnosis of Gestational Diabetes Mellitus in Southern Sweden

**DOI:** 10.1155/2016/8905474

**Published:** 2016-12-26

**Authors:** Anastasia Katsarou, Rickard Claesson, Claes Ignell, Nael Shaat, Kerstin Berntorp

**Affiliations:** ^1^Department of Clinical Sciences, Lund University, Malmö, Sweden; ^2^Department of Endocrinology, Skåne University Hospital, Malmö, Sweden; ^3^Department of Obstetrics and Gynaecology, Office for Healthcare “Kryh”, Ystad, Sweden; ^4^Department of Obstetrics and Gynaecology, Office for Healthcare “Sund”, Helsingborg, Sweden

## Abstract

*Aim*. The aim of this study was to examine seasonal patterns in glucose tolerance and in the diagnosis of gestational diabetes mellitus (GDM).* Methods*. Altogether, 11 538 women underwent a 75-g oral glucose tolerance test (OGTT) in the twenty-eighth week of pregnancy during the years 2003–2005 in southern Sweden. GDM was defined by the 2-h capillary glucose concentration in the OGTT (≥8.9 mmol/L). Chi-squared test, analysis of variance, and regression analyses were used for statistical evaluations.* Results*. The seasonal frequency of GDM ranged from 3.3% in spring to 5.5% in summer (*p* < 0.0001). Mean 2-h glucose concentrations followed the same seasonal trend, with a difference of 0.15 mmol/L between winter and summer (*p* < 0.0001). The 2-h glucose level increased by 0.009 mmol/L for every degree increase in temperature (*p* < 0.0001). In regression analysis, summer (June–August) was associated with increased 2-h glucose level (*p* < 0.001) and increased frequency of GDM compared to the other seasons (odds ratio 1.51, 95% confidence interval 1.24–1.83, and *p* < 0.001).* Conclusions*. Our findings suggest seasonal variation in the 2-h glucose concentration in the OGTT and in the proportion of women diagnosed with GDM, with a peak in the summer.

## 1. Introduction

While seasonality in the onset of type 1 diabetes is well documented [1], less is known about seasonality in the diagnosis of type 2 and gestational diabetes mellitus (GDM). Doró et al. (2006) reported an increased incidence of type 2 diabetes onset in winter [2]. A similar pattern in HbA1c and glucose levels has been reported in diabetes patients, possibly because of seasonal variations in environmental conditions, such as diet and exercise [3–6]. In contrast, Schmidt et al. (1994) reported a 4-fold increase in the frequency of GDM in the summer compared with the winter, which they related to increased 2-h glucose levels in the oral glucose tolerance test (OGTT) at higher ambient temperatures [7]. Similar results of temperature-induced differences in postload venous glucose levels have previously been reported by Akanji et al. in subjects with and without diabetes [8, 9]. However, two other studies from Australia and the UK found no clinically significant evidence of any seasonal variation in the prevalence of GDM or the 2-h glucose levels in the OGTT [10, 11].

The prevalence of GDM in a given population depends on the screening approach and on the diagnostic criteria used [12]. In southern Sweden, universal screening for GDM with a 75-g OGTT has been performed at the antenatal clinics since 1995, with no major changes in the diagnostic procedure. The diagnosis of GDM is based on the 2-h capillary glucose concentration [13]. During the years 2003–2005, pregnant women, representing different glucose categories according to the OGTT, were invited to take part in a follow-up study postpartum, the Mamma Study. During the period of recruitment, a large number of test results were made available and they form the basis of the present study. The aim was to determine whether there were any differences in glucose tolerance by season and, consequently, if the frequency of GDM showed any seasonal variation. Such differences are important to elucidate, since they may affect the diagnostic procedure and interpretation of the results.

## 2. Materials and Methods

### 2.1. GDM Screening

The screening programme for GDM in southern Sweden has been described in detail elsewhere [13]. Briefly, a 75-g OGTT is offered to all women in the twenty-eighth week of gestation after overnight fasting and also in week 12 if there is a history of GDM in previous pregnancies or a first-degree relative with diabetes. The HemoCue blood glucose 201+ system (HemoCue AB, Ängelholm, Sweden) is used to perform immediate analysis of capillary glucose concentrations, which are reported as equivalent plasma glucose concentrations [14]. To ascertain the quality of the individual testing, double sampling is used, with acceptance of a divergence of ≤0.3 mmol/L. If this is not reached, a third sample is taken, and if the divergence between two of the samples is still not acceptable, the equipment is checked and the OGTT is not regarded as being valid.

The diagnostic criteria for GDM used in the present study are a slight modification of those recommended by the World Health Organization in 1999, defining GDM as a 2-h venous plasma glucose concentration of ≥7.8 mmol/L, corresponding to a capillary plasma glucose concentration of ≥8.9 mmol/L [15]. The diagnosis was based on the mean of the two measurements.

### 2.2. Study Population

Recruitment to the Mamma Study took place during the years 2003–2005 and involved four of the five delivery departments in the county of Skåne in southern Sweden [16]. During the recruitment period, OGTT results from the local antenatal clinics were sent to the study coordinator, enabling identification of the test results of women who consented to be enrolled; it also ensured correct sampling technique [13]. Initially, 11 976 OGTT results in total were reported. If a woman had more than one pregnancy during the study period, only the first one was included. Likewise, if a woman underwent more than one OGTT during the same pregnancy, only the one performed in pregnancy week 28 was included. This restricted data set (*n* = 11 538) formed the basis of the present evaluation. Mean monthly temperatures during the study period were obtained from the Swedish Meteorological and Hydrological Institute (http://opendata-download-metobs.smhi.se/explore/?parameter=3#).

The study was carried out in accordance with the Declaration of Helsinki. Written informed consent was obtained from all participants, and the study protocol was approved by the Ethics Committee of Lund University (LU 259-00).

### 2.3. Statistical Analysis

OGTT results from the 3-year study period were grouped together into months and seasons (winter: December–February; spring: March–May; summer: June–August; autumn: September–November). Chi-squared test was used to test for differences in frequencies between months and seasons, and one-way analysis of variance (ANOVA) was used to test for the corresponding differences in means. Multivariable logistic regression was used to examine whether month or season was associated with the diagnosis of GDM and multivariable linear regression was used to examine the corresponding associations with 2-h glucose levels. The relationship between mean monthly temperatures and mean monthly 2-h glucose concentrations was evaluated by simple linear regression.

IBM SPSS Statistics 22 for Windows (IBM Corporation, Armonk, NY) was used for analysis. Two-sided *p* values of less than 0.05 were considered to be statistically significant.

## 3. Results

Of the 11 538 women who underwent an OGTT during the study period, a total of 487 women (4.2%) were diagnosed with GDM.

Table 1 shows the study material, organized by month and season. The monthly frequency of GDM ranged from 2.9% in March to 5.8% in June, and the seasonal frequency of GDM ranged from 3.3% in spring to 5.5% in summer. The differences in frequencies were statistically significant, both for month (*p* = 0.01) and for season (*p* < 0.0001). The mean age of participating women was 29.9 (standard deviation 5.1) years, and the ages ranged from 15 to 49 years. The age of the women differed statistically significantly between months and seasons (*p* < 0.001). However, no significant differences in the monthly distributions of age were noted.

Mean monthly temperature ranged from −0.6°C in winter to 17.7°C in summer (Table 1). In a simple linear regression with 2-h plasma glucose as the dependent variable and mean monthly temperature as the predictor variable, the coefficient in the equation was 0.009, suggesting that the 2-h glucose level increased by 0.009 mmol/L for every degree increase in temperature (*p* < 0.0001).

**Figure 1:**
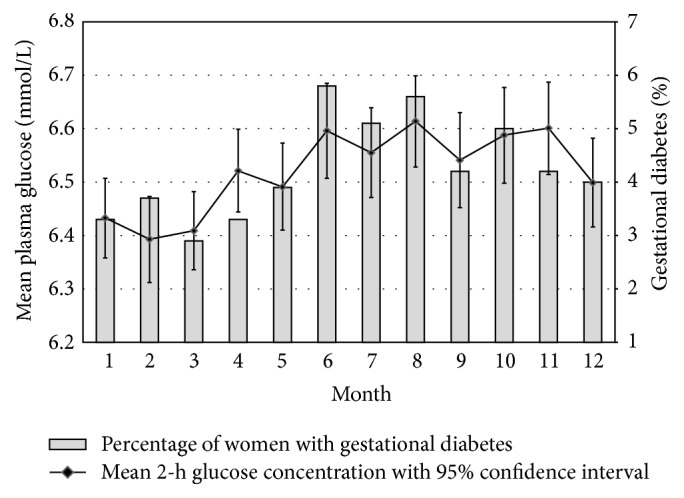
Monthly mean 2-h glucose levels and the monthly percentage of women with gestational diabetes mellitus.

Figure 1 illustrates the monthly mean 2-h glucose level during the OGTT (with 95% confidence interval (CI)) and the monthly percentage of women with GDM. Though numerically small, the differences in 2-h glucose levels were statistically significant (*p* < 0.001), with the lowest values observed from January to March and peak levels from June to August. A similar seasonal trend was seen for the percentage of women with 2-h glucose levels in the GDM range (2-h glucose level ≥ 8.9 mmol/L). There were no differences in the distribution of glucose concentrations between months or seasons.

In regression analysis, adjusting for age, the summer months (June to August) were found to be associated with increased 2-h glucose levels (*p* < 0.001) and increased frequency of GDM compared to all other months (OR = 1.51 (95% CI: 1.24–1.83); *p* < 0.001).

## 4. Discussion

In this observational study of 11 538 pregnancies, we found seasonal variations in the 2-h glucose level in the OGTT performed in the twenty-eighth week of gestation, giving seasonal variations in the percentage of women diagnosed with GDM—with a peak in the summer.

Until recently, there have only been three previous studies in the literature evaluating seasonality in GDM [7, 10, 11]. Using a 2-h venous plasma glucose concentration of ≥7.8 mmol/L to define GDM during a standardized 75-g OGTT in 1 113 consecutively tested women in Brazil, Schmidt et al. (1994) reported that the frequency of GDM varied in relation to the ambient temperature, from 4.8% in winter to 14.8% in summer [7]. For every degree increase in temperature, the mean 2-h glucose level increased by 0.07 mmol/L, while the fasting glucose levels were unaffected by temperature. Standardizing the results at 23°C indicated that women were overdiagnosed by 19% at higher temperatures and underdiagnosed by 45% at lower temperatures [7]. Similar results have been described in small-scale studies outside of pregnancy [8, 9]. Increased arterialisation of the venous blood at elevated temperatures has been suggested to be a plausible explanation [17]. Whether these variations in glucose levels result from an acute effect of temperature rather than a chronic one is not fully understood, although some experimental studies have indicated an acute effect [18, 19]. Interestingly, in a very recent study from the coastal area of Australia, Moses et al. reported significantly lower median 1-h and 2-h glucose levels in the OGTT in the winter compared with the overall 1-h and 2-h results, when evaluated in a cohort of 7 369 pregnant women prospectively followed up during a 3-year period [20]. Furthermore, in a population-based study from South Australia, Verburg et al. recently reported seasonal variation in the diagnosis of GDM based on the estimated date of conception [21].

Since the present study was based on capillary glucose measurements, previous findings cannot be directly extrapolated to results using our methodology. However, temperature-induced changes in peripheral blood flow may very well affect the composition of capillary blood as well, representing a mixture of arterial and venous blood. According to national statistics, the average temperature in the region varied between −0.6°C in winter and +17.7°C in summer during the study period. With respect to the simple linear regression analysis, indicating that for every one degree increase in temperature the glucose concentration increased by 0.009 mmol/L, this difference in temperature between summer and winter corresponds to a difference of 0.15 mmol/L in glucose concentration. It is important to note that OGTT as such has a rather low reproducibility, especially for 2-h glucose levels in the intermediate range [22–24]. This means that a difference of 0.15 mmol/L would have diagnostic implications in women with glucose concentrations close to the diagnostic limit, thereby increasing the frequency of GDM in the summer.

Though not regarded as a diagnostic standard [25], capillary finger-tip tests have been used in the screening programme for GDM in southern Sweden ever since they were first introduced in 1995 [13]. For practical reasons, this was regarded as a prerequisite when introducing the OGTT on a large scale. Glucose measurements based on capillary samples are generally believed to show greater variation than venous glucose measurements [26]. If the hand is cold there is a risk of squeezing and “milking” of the finger, increasing the proportion of extracellular fluid, resulting in a lower glucose concentration. However, since glucose measurements in the present study were based on measurements after two hours at room temperature in the antenatal clinic, such an explanation would seem less likely.

In contrast to our findings and those of Schmidt et al. [7], Janghorbany et al. (2006) could not prove any seasonality in glucose tolerance or in the incidence of GDM in 4 942 pregnancies in Plymouth, UK [11]. However, it is important to note that Plymouth has a very mild climate, with little seasonal variation. Furthermore, only 11% (*n* = 539) of the women underwent an OGTT during pregnancy and the incidence figures were based on those with an abnormal OGTT (*n* = 90), as opposed to all the others who either had a normal OGTT or initially screened negative by random plasma glucose testing or risk factors. In a study from Australia, Moses and Griffiths (1995) examined seasonal trends in 2 749 women consecutively tested with a 75-g OGTT [10]. Interestingly, after adjustments for various risk factors, multiple regression analysis revealed a significant association between the monthly temperature and the 2-h glucose level in the OGTT; the 2-h glucose level increased by 0.026 mmol/L for every degree increase in temperature [10]. However, Chi-squared analysis did not suggest any seasonal variation in the diagnosis of GDM, and it was concluded that the association between 2-h glucose and temperature was unlikely to be of any clinical importance in the temperate coastal area of Australia, with the mean monthly temperature during the study period ranging from 13.6°C in July to 22.3°C in January [10].

Our results do not agree with previous observations by Doró et al. from Hungary, of an increased incidence of type 2 diabetes in winter, although it should be noted that the incidence figures reported were based on the initiation of antidiabetic therapy in individuals with previously diagnosed diabetes, therefore not representing the “true” diabetes onset [2]. Likewise, worsening of metabolic control in subjects with type 2 diabetes in winter has been described in a number of studies [3–6]. Since diet and exercise are hallmarks of the treatment of type 2 diabetes, it is reasonable to assume that environmental factors, such as diet and exercise patterns, have an important role in the seasonal variation in glucose metabolism in patients with diabetes. Seasonal variation in the diagnosis of GDM possibly reflects seasonality of environmental influences early in gestation, during placental development, affecting placental metabolism and glucose homeostasis later on in pregnancy [21]. Many factors vary with season, including the nutritional quality of foods, temperature, the number of hours of sunshine, and vitamin D synthesis. Maternal vitamin D deficiency in early pregnancy has been associated with increased risk of GDM [27]. Moreover, seasonal variation in vitamin D status, quantified as the total number of hours of sunshine during the three months preceding the onset of diabetes, was suggested as an explanation for the seasonality of type 2 diabetes reported by Doró et al. [28].

The strength of the present study was the uniform diagnostic procedure for GDM, based on universal screening with a 75-g OGTT and enabling identification of a large cohort of women in one particular week of gestation. Unfortunately, we did not have access to individual data other than age, so the figures are crude and unadjusted for other potentially important risk factors, such as body mass index and ethnicity [29]. However, we have no reason to believe that this would have any major effect on the results, due to the size of the material and the exclusive use of OGTT. Another weakness of the study was that we only had information on mean monthly temperatures during the study period and not the mean temperature on specific days, which makes it difficult to draw any firm conclusions on the effect of temperature on our results.

We have previously shown that approximately 5-6% of women with GDM in our region have positive islet cell autoantibodies as a marker of autoimmune pathogenesis and therefore of type 1 diabetes [30, 31]. Of these, 50% had developed type 1 diabetes within ten years postpartum [30]. Seasonality in the incidence of type 1 diabetes has been described [1], but this is probably not of any relevance to the present findings, due to the overall low proportion of women who would be expected to have autoimmune diabetes in our material.

## 5. Conclusions

Based on a universally performed OGTT in the twenty-eighth week of pregnancy, seasonality in the proportion of women diagnosed with GDM was observed, with a peak in the summer. The mean 2-h glucose concentration in the OGTT followed the same seasonal trend. The findings may be related to the increased ambient temperature in the summer. Further studies are needed to determine whether our results are reproducible and if they are, to investigate the cause(s) of seasonality, as such variations may have implications for the diagnostic procedure and for interpretation of the results.

## Figures and Tables

**Table 1:** Description of the study material, organized by month and season.

	OGTT *n*	GDM *n* (%)	2-h glucose, mmol/L Mean (SD)	Age, years, mean (SD)	Temperature, °C, mean
*Month*					
January	1 094	36 (3.3)	6.4 (1.3)	30.4 (5.0)	0
February	928	34 (3.7)	6.4 (1.3)	30.1 (5.3)	−0.6
March	1 082	31 (2.9)	6.4 (1.2)	30.1 (5.2)	2.4
April	1 027	34 (3.3)	6.5 (1.3)	30.1 (5.0)	7.5
May	1 057	41 (3.9)	6.5 (1.4)	30.4 (5.1)	12.1
June	1 009	59 (5.8)	6.6 (1.4)	29.8 (5.1)	15.2
July	974	50 (5.1)	6.6 (1.3)	30.0 (5.0)	17.7
August	928	52 (5.6)	6.6 (1.3)	29.6 (5.0)	17.6
September	781	33 (4.2)	6.5 (1.3)	29.5 (5.3)	14.4
October	835	42 (5.0)	6.6 (1.3)	29.6 (5.1)	8.4
November	897	38 (4.2)	6.6 (1.3)	29.6 (5.2)	5.3
December	926	37 (4.0)	6.5 (1.3)	29.8 (5.1)	2.9
*Season*					
Winter	2 948	107 (3.6)	6.4 (1.3)	30.1 (5.1)	0.7
Spring	3 166	106 (3.3)	6.5 (1.3)	30.2 (5.1)	7.3
Summer	2 911	161 (5.5)	6.6 (1.4)	29.8 (5.0)	16.8
Autumn	2 513	113 (4.5)	6.6 (1.3)	29.6 (5.2)	9.2

GDM, gestational diabetes mellitus; OGTT, oral glucose tolerance test; SD, standard deviation.
